# Items

**Published:** 1875-05

**Authors:** 


POSTPONED.—The time of meeting of the Ohio State Medical Society has been changed from the second to the third Tuesday in June. The meeting will be held at Put-in-Bay, Tuesday, June 15th, 1875.——ZIEMSSEN'S CYCLOPÆDIA.—Messrs. Wm. Wood & Co., the publishers of this work wish us to announce that it will not be sold in *separate volumes*, but only in complete sets. Those wishing the work are therefore cautioned against purchasing any odd volumes which may be offered for sale.——ENTERPRISING.—The American Medical Weekly, of Louisville, Dr. E. S. Gaillard, Editor, had a complete report of the proceedings of the American Medical Association in its issue of May 8th. Our thanks are due the Editor for an advance proof sheet.——MERCURY IN SYPHILIS.—From the *Doctor* we learn that Dr. Drysdale who was at one time an active opponent of the use of mercury in syphilis has changed his views, and now believes that in many cases it may be used with benefit.——THE ASSOCIATION OF AMERICAN MEDICAL EDITORS at its annual meeting in Louisville, selected the following officers: President, Dr. Bell of the *Sanitarian;* Vice President, Dr. Wood, Jr., of the *Philadelphia Medical Times;* Secretary, Dr. F. C. Davis, of the *Chicago Medical Times;*——Dr. D. W. CHEEVER has been appointed Professor of Clinical Surgery at Harvard Medical College.——ERIE COUNTY MEDICAL SOCIETY.—The Semi-Annual Meeting of the Erie County Medical Society will be held Tuesday, June 8th, in the Medical College. Matters of importance are expected to come before the Society.

